# Who Became Lonely during the COVID-19 Pandemic? An Investigation of the Socioeconomic Aspects of Loneliness in Japan

**DOI:** 10.3390/ijerph19106242

**Published:** 2022-05-20

**Authors:** Mostafa Saidur Rahim Khan, Pattaphol Yuktadatta, Yoshihiko Kadoya

**Affiliations:** School of Economics, Hiroshima University, 1-2-1 Kagamiyama, Higashihiroshima 7398525, Japan; d206295@hiroshima-u.ac.jp (P.Y.); ykadoya@hiroshima-u.ac.jp (Y.K.)

**Keywords:** loneliness, COVID-19 pandemic, socioeconomic attributes, subsample analysis, Japan

## Abstract

The COVID-19 pandemic has impacted social and economic aspects of people’s lives in different ways, causing them to experience different levels of loneliness. This study examines the extent of loneliness among men and women of various ages in Japan during the pandemic and attempts to determine the underlying causes. We used data from Hiroshima University’s nationwide survey conducted before and during the pandemic in Japan. The sample consists of 3755 participants, of which 67% are men and 33% are women with an average age of 51 years (SD = 13.64). Using mean comparison tests and probit regression models, we show that loneliness is a common occurrence among the Japanese population and that a significant number of people became lonely for the first time during the pandemic. In general, loneliness was greater among younger respondents, but older people became lonelier during the pandemic. Simultaneously, we observed significant differences in loneliness across age and gender subsamples. Although depression and subjective health status contributed to loneliness, we found no single explanation for the loneliness experienced by people during the pandemic; rather, subsample analysis revealed that the causes of loneliness for each group differed. Nevertheless, we discovered that older people are at a higher risk of developing loneliness during the pandemic due to a variety of socioeconomic and behavioral factors. The findings of this study suggest that health authorities should not generalize cases of loneliness, but rather intervene individually in each group to avoid further complications.

## 1. Introduction

The COVID-19 pandemic has had such a profound impact on people’s social, economic, and personal lives that their psychological well-being has severely deteriorated [1]. Loneliness, one of the components of social psychology, has been affected as a result of changing socioeconomic situations, the maintenance of social distance and the health safety measures, and the enormity of health- and livelihood-related concerns [2,3]. Furthermore, the government and society’s responses to the crisis have impacted people’s psychological stress and contributed to their loneliness [4]. Several longitudinal studies investigated the actual change in psychological stress, depression, and anxiety during the pandemic and found a deterioration in psychological conditions [5,6,7]. However, several studies also found that the pandemic has not deteriorated the depression, anxiety, and stress of people [8,9,10]. Loneliness, a global phenomenon that has been longstanding, has been exhibited extensively among various socioeconomic groups during the pandemic. Similar to other psychological distress, the evidence of heterogeneity and inconsistency in the magnitude of loneliness and its causes, particularly with regard to age and gender, necessitates further investigation. For example, Khan and Kadoya [2], Weissbourd et al. [11], Ausín et al. [12], and Bu et al. [13] found increasing loneliness among people during the pandemic, while Luchetti et al. [14], McGinty et al. [15], and Peng and Roth [16] found a steady level of loneliness. Weissbourd et al. [11], Wickens et al. [17], Bu et al. [13], and Li and Wang [18] evidenced that younger people became lonelier during the pandemic, while Khan and Kadoya [2] and Luchetti et al. [14] reported slightly higher loneliness among older people. Tillberg et al. [19] and Kucharska-Newton et al. [20], in particular, provided evidence of an increasing loneliness among the older people. Furthermore, Wickens et al. [17], Khan and Kadoya [2], Seifert and Hassler [21], and Bu et al. [13] discovered that women were more vulnerable to loneliness, but Stickley and Ueda [22] discovered that men were more likely to be lonely. Groarke et al. [23], on the other hand, discovered that there was no significant gender difference in loneliness during the pandemic. The heterogeneity and inconsistency are not entirely unexpected given the nature of the problem and the socioeconomic conditions in which people live. Moreover, such heterogeneity and inconsistency have been observed in the pre-pandemic loneliness literature too [24,25,26]. Thus, a follow-up study involving gender- and age-based subsamples will help to advance existing efforts to understand the degree of loneliness and related risk factors during the pandemic. Moreover, Dahlberg [4] identified several methodological difficulties in existing studies concerning data collection, for which additional studies on diverse socioeconomic groups could provide a solution. Thus, it is important to study the extent of loneliness in various gender- and age-based subsamples of the Japanese population during the pandemic, with the goal of elucidating the underlying causes.

It is important to understand why the pandemic may contribute to loneliness differently to different socioeconomic groups. Dahlberg [4] summarized theoretical discussions on loneliness by presenting two perspectives on loneliness, namely the cognitive perspective and the resource perspective, which appear to be valid during the pandemic as well. From the cognitive perspective, loneliness can be defined as the lack of social attachment or loss of social contact that impacts people’s emotional conditions [27]. Due to social isolation, working from home, job loss, transit restrictions, and other factors, social contact has been substantially disrupted during the pandemic. Importantly, such loss of social contact is observed in varying degrees across socioeconomic groups, prompting them to suffer loneliness at different levels. After all, people had a different social engagement prior to the pandemic and have been disproportionately affected during the pandemic. For example, Kotwal et al. [28] observed that social isolation increased emotional loneliness, but van Tilburg et al. [19] found that many people experienced increased emotional loneliness during the pandemic even without social isolation. According to van Tilburg et al. [19], emotional loneliness can be caused by personal loss, anxieties, and a lack of trust in social institutions. Lack of social integration may not affect everyone equally and cause loneliness. During the pandemic, many people have reduced their social expectations and gained an understanding of the hardships of others, which works as a buffer against loneliness [14,19]. From the resource perspective, loneliness may be influenced by access to individual resources such as communication and social skills, as well as contextual material resources, such as socioeconomic status, health, and neighborhood status, all of which explicitly influence people’s social relations [27,29,30,31]. Thus, according to this viewpoint, loneliness among people from a specific socioeconomic category is determined by the resources they have or the loss of resources they faced during the pandemic. Previous studies support this perspective by discovering an association between loneliness and income [32], physical and mental health status [28,33], individuals living alone [19,34], and lack of social contacts [19,35].

Japan is a prominent ageing nation, with many elderly individuals highly vulnerable to loneliness as a result of living alone and a lack of attention from family members due to changing socioeconomic conditions. Japanese youth experienced different levels of loneliness during the pandemic due to a lack of social contact, job loss, and the resulting over usage of digital devices. Furthermore, loneliness is also influenced by the long-nurtured Japanese traditions and culture. Being a collectivist society, Japan considers social engagement as a core value of daily life. This habituates Japanese people to higher levels of social engagement, making them more vulnerable to loneliness during social isolation. A sudden disruption in social lives is likely to affect people’s psychological wellbeing, particularly those with pre-existing mental health conditions such as depression and anxiety [36,37]. Given that many of Japan’s prefectures were in a state of emergency for an extended period of time, it is expected that the Japanese people experienced loneliness to varying degrees depending on the extent of their loss of social contact. In Japan, relatively little research has been conducted on the risk of loneliness and its associated negative effects, with a particular emphasis on population diversity. Thus, a comprehensive study of loneliness is required in Japan to understand the nature of loneliness among various socioeconomic groups and to identify gender- and age-specific risk factors that may have an adverse effect on mental health. This study examines loneliness among men and women of various age groups during the pandemic in Japan, with the goal of elucidating the underlying causes. In particular, we focused on the samples that became lonely during the pandemic. Our study contributes to the existing body of literature in at least two ways. First, we provide detailed evidence on loneliness among men and women of various ages during the pandemic with a particular focus on those who became lonely during the pandemic but were not lonely before. Second, we show that the risk factor for loneliness varies significantly across age and gender subsamples.

## 2. Data and Methods

### 2.1. Data

Our study uses the panel dataset from the Hiroshima University’s Household Behavioral and Financial Survey, which was created by Nikkei Research, a leading research company with extensive databases reflecting the socioeconomic status of the Japanese population. The database, which adheres to the random sampling procedure while maintaining representativeness, contains information on the preferences and socioeconomic status of Japanese adults from 2020 and subsequent years. The database reflects the COVID-19 pandemic’s time frame. The first round of data collection was conducted between 20 and 25 February 2020, at the start of the COVID-19 pandemic or before the WHO declared COVID-19 a global pandemic on 11 March 2020 [38]. The first dataset includes 17,463 samples. The second round of data collection was conducted during the pandemic (19–26 February 2021), and 6103 of the original samples chose to participate. Regrettably, some information on household financial status is missing. We excluded 2348 observations as our study sought to investigate the relationship between loneliness and socioeconomic status. As a result, the total number of observations in our final merged dataset consists of 3755.

According to Hiroshima University’s ethics committee, this study does not require ethical scrutiny. Although we investigated the effect of COVID-19 on loneliness, this is a socioeconomic study. It contains no intrusive or sensitive information that could lead to identity recognition. Nevertheless, we informed the respondents about the survey’s purpose and obtained their consent.

### 2.2. Variable Definitions

“Loneliness”, the dependent variable of our study, was measured following the UCLA methodology [39], which included three questions such as “How often do you feel a lack of companionship”, “How often do you feel left out”, and “How often do you feel isolated from others”. The options to respond to these questions were “Hardly ever or never”, “Some of the time”, and “Often”. We classified respondents as lonely (Loneliness = 1) if they frequently/occasionally felt “a lack of companionship”, “left out”, or “isolated from others”. However, if respondents rarely or never felt any of the aforementioned emotions, we classified them as not lonely (Loneliness = 0). Given that our study focuses on the effect of the pandemic on loneliness, our second dependent variable “Became lonely” observed the change in loneliness over the course of a year during the pandemic. We classified respondents as becoming lonely (Became lonely = 1) if they had little or no loneliness at the beginning of the pandemic but became lonely a year later. Otherwise, we classified the respondents into two groups: those who did not become lonely and those who were already lonely (Became lonely = 0).

We used the 2020 dataset to obtain respondents’ demographic characteristics, such as gender, child-rearing status, residence, and years of education, as explanatory variables. We also included socioeconomic variables from both datasets, such as age, marital status, living status, employment status, and household financial status. Furthermore, we used the 2020 dataset to include the financial literacy variable, which is a proxy variable for rational financial and health behaviors [40,41,42,43,44,45]. We also included subjective assessments of health-related issues, such as health status and depression, as well as other variables, such as future anxiety, financial satisfaction, and a myopic view of the future. The detailed definitions of the main variables are provided in Table 1.

### 2.3. Descriptive Statistics

The main variables of this study are described in Table 2. The results show that 74% of respondents in 2021 were lonely, of which 12% became lonely during the pandemic. Results also reveal that 67% of the respondents were male, with an average age of 50.99 years, have 15 years of education, and attained a financial literacy score of 0.68. Respondents’ family structure shows that 20% were living alone, 67% had a spouse or partner, and 58% had children. Moreover, 58% of respondents resided in rural areas and 64% were employed.

For financial status, the results show that respondents had an average annual household income of 6.46 million yen and household assets of 21.0 million yen. Regarding the subjective assessment on various issues, the results show that on a five-point scale, respondents had an average financial satisfaction score of 2.77, subjective health status score of 3.26, depression score of 2.95, future anxiety score of 3.69, and myopic view of the future score of 2.67.

We divided the entire sample into subsamples based on gender and age. Table 3 and Table 4 provide additional descriptions of loneliness and becoming lonely, respectively. Table 3 demonstrates that loneliness varies by age group and gender at the 99% significance level, whereas Table 4 shows that becoming lonely varies by gender the 99% significance level. Although we found no difference in the “Became Lonely” variable among females of different ages, we found the difference in the variable among males of different ages at the 95% significance level.

### 2.4. Methods

We use the following equations to analyze the relationship between loneliness and the explanatory variable:

(1) $$Y_{1i}=f\left(X_{i},\varepsilon_{i}\right)$$

(2) $$Y_{2i}=f\left(X_{i},\varepsilon_{i}\right)$$

(3) $$Y_{2i}=f\left(X_{i},∆X_{i},\varepsilon_{i}\right)$$

where $Y_{1}$ is loneliness, $Y_{2}$ is became lonely, X is a vector of demographic, socio-economic, and behavioral variables, and ∆X is a vector of change in various demographic, socio-economic, and behavioral variables during the pandemic, such as change in marital status, change in living conditions, change in employment status, change in household income and assets, change in health status, future anxiety, satisfaction, depression, and myopic view of the future. The changed variables reflect the difference in values before and during the pandemic. Since our dependent variables are binary, we performed weighted logit regression to estimate the equations.

Furthermore, we performed a multicollinearity test, as our regression results are vulnerable to a multicollinearity problem (reports are available upon request). According to our findings, the variance inflation factors of the explanatory variables are less than 10. Therefore, our regressions are unlikely to exhibit multicollinearity. We also conducted weighted regression, as our study may have sample representation issues in addition to the multicollinearity issue.

The full model specifications for Equations (1)–(3) are as follows:


(4) $$\begin{array}{cc} {\mathrm{Loneliness}}_{i}=\beta_{0} & +\beta_{1}{\mathrm{male}}_{i}+\beta_{2}{\mathrm{age}}_{i}+\beta_{3}{\mathrm{spouse}}_{i}+\beta_{4}{\mathrm{children}}_{i}\\ & +\beta_{5}{living alone}_{i}+\beta_{6}{living in rural}_{i}+\beta_{7}{\mathrm{education}}_{i}\\ & +\beta_{8}{\mathrm{employed}}_{i}+\beta_{9}\log({household income}_{i})\\ & +\beta_{10}\log({household assets}_{i})+\beta_{11}{financial literacy}_{i}\\ & +\beta_{12}{subjective health}_{i}+\beta_{13}{future anxiety}_{i}\\ & +\beta_{14}{financial satisfaction}_{i}+\beta_{15}{\mathrm{depression}}_{i}\\ & +\beta_{16}{myopic view of the future}_{i}+\varepsilon_{i} \end{array}$$

(5) $$\begin{array}{cc} {Became lonely}_{i} & =\beta_{0}+\beta_{1}{\mathrm{male}}_{i}+\beta_{2}{\mathrm{age}}_{i}+\beta_{3}{\mathrm{spouse}}_{i}+\beta_{4}{\mathrm{children}}_{i}\\ & \;+\beta_{5}{living alone}_{i}+\beta_{6}{living in rural}_{i}+\beta_{7}{\mathrm{education}}_{i}\\ & \;+\beta_{8}{\mathrm{employed}}_{i}+\beta_{9}\log({household income}_{i})\\ & \;+\beta_{10}\log({household assets}_{i})+\beta_{11}{financial literacy}_{i}\\ & \;+\beta_{12}{subjective health}_{i}+\beta_{13}{future anxiety}_{i}\\ & \;+\beta_{14}{financial satisfaction}_{i}+\beta_{15}{\mathrm{depression}}_{i}\\ & \;+\beta_{16}{myopic view of the future}_{i}+\varepsilon_{i} \end{array}$$

(6) $$\begin{array}{cc} {Became lonely}_{i} & =\beta_{0}+\beta_{1}{\mathrm{male}}_{i}+\beta_{2}{\mathrm{age}}_{i}+\beta_{3}{recently divorced}_{i}+\beta_{4}{\mathrm{children}}_{i}\\ & \;+\beta_{5}{became alone}_{i}+\beta_{6}{living in rural}_{i}+\beta_{7}{\mathrm{education}}_{i}\\ & \;+\beta_{8}{left employment}_{i}+\beta_{9}\log({change in household income}_{i})\\ & \;+\beta_{10}\log({change in household assets}_{i})\\ & \;+\beta_{11}{financial literacy}_{i}+\beta_{12}{change in health status}_{i}\\ & \;+\beta_{13}{change in future anxiety}_{i}\\ & \;+\beta_{14}{change in financial satisfaction}_{i}\\ & \;+\beta_{15}{change in depression}_{i}+\beta_{16}{change in the myopic view}_{i}\\ & \;+\varepsilon_{i} \end{array}$$

## 3. Results

### 3.1. Loneliness during the Pandemic

Table 5 presents the regression results for the total sample’s loneliness during the pandemic. The estimates from the full model specification are presented in the last column. Age, children, income, assets, and health status were found to be negatively associated with loneliness during the pandemic. Furthermore, people with a high level of future anxiety and depression were more likely to feel lonely. In addition to finding an association between loneliness and the explanatory variables, we used the add-drop variables method to test the robustness of our findings, the result of which are shown in the first three columns. Overall, most of our estimates were found to be robust and consistent.

Table 6 presents the regression results for the loneliness of gender and age subsamples during the pandemic. Except for the depression variable, we discovered that the associations vary across age groups and gender. People suffering from depression, regardless of age and gender, were found to be more likely to feel lonely.

We found that males under the age of 36 who have children, a higher household income, and live alone are less likely to feel lonely during the pandemic. Moreover, those who live in rural areas and have higher household assets values are more likely to feel lonely. We also discovered that men aged between 36 and 50, with better health status and higher financial satisfaction, are less likely to feel lonely. However, those living alone and having a more myopic view of the future are more likely to feel lonely. Furthermore, we found a negative association between loneliness and age, as well as loneliness and subjective health in men aged between 51 and 65. We also discovered that retired men (aged above 65), with good health and higher income, are less likely to feel lonely, while those with a spouse or living alone are more likely to feel lonely.

In the female samples, we found that females aged between 36 and 50, who live in rural Japan, and have better health are less likely to feel lonely. However, among retired female samples, we found a negative association between age and loneliness, as well as subjective health and loneliness. We also discovered that retired women with higher financial literacy, higher financial satisfaction, and a more myopic view of the future are more likely to suffer from loneliness. Furthermore, women aged between 51 and 65 who have children are less likely to suffer from loneliness.

### 3.2. Who Became Lonely during the Pandemic?

The regression results of Equation (2) are presented in Table 7. It demonstrates the relationship between those who became lonely during the pandemic and the explanatory variables. Similar to Table 5, the estimates belonging to the full model specification are provided in the last column, whereas the robustness checks are presented in the first three columns. Overall, most of our estimates are robust and consistent. The only statistically significant association we discovered was between gender and loneliness. We found that men are more likely to experience loneliness during the pandemic.

To gain more insight, we conducted a subsample analysis by age group and gender, the results of which are presented in Table 8. Among the male subsamples, males under the age of 36 who are older, married, and in better health are more likely to feel lonely during the pandemic. The relationship between loneliness and marital status was also observed among males aged between 36 and 50, as well as among those aged above 65. Furthermore, men aged over 65 years with higher financial literacy were also found to be more likely to become lonely during the pandemic. Among the female subsamples, we found that women under the age of 36 who have a spouse and are depressed are less likely to become lonely during the pandemic. Those with children and a job, on the other hand, are more likely to become lonely. Females aged between 36 and 50 who live alone are more likely to become lonely, while those with more financial literacy, better health, and worsening depression are less likely to become lonely. Furthermore, among women aged between 51 and 65, we discovered positive associations between loneliness and age, spouse, and financial literacy variables. We also discovered that women with a greater level of education and household assets are less likely to be lonely. Among retired women, those who have a spouse and a high assets value are less likely to be lonely, but those with a higher education, a higher income, worsening depression, and a more myopic view of future are more likely to become lonely during the pandemic.

Table 9 shows the regression results of Equation (3), which estimates the relationship between changes in marital status, living condition, employment status, household income and assets, health status, future anxiety, satisfaction, depression, and myopic view of the future, as well as basic demographic variables. Our estimations have shown to be reliable and consistent. According to our findings, women are more likely to experience loneliness during the pandemic.

The regression results of the subsample analysis are presented in Table 10. Unfortunately, we had to exclude several variables owing to the perfect multicollinearity problem.

Among the male subsamples, men below the age of 36 were found to be more likely to experience loneliness during the pandemic. Moreover, the higher likelihood of becoming lonely during the pandemic are found among recently divorced men aged between 51 and 65, financially literate men aged 65 and above, men aged between 36 and 50 with lower subjective health status, and older men with worsening depression.

Among the female subsamples, we found that women above the age of 51 are more likely to become lonely, whereas those who under the age of 36 and became alone during the pandemic are more likely to become lonely. Moreover, a higher likelihood of becoming lonely during the pandemic are found among less-educated women aged between 51 and 65, women aged between 36 and 50 who left job and with lower subjective health status, and younger women with worsening depression.

## 4. Discussion

Loneliness does not only refer to living alone or lack of social contact, it also refers to the subjective experience of having inadequate social relationships even when with family [46,47,48]. Although loneliness has long been a prevalent phenomenon among a larger section of the population, the emergence of the COVID−19 pandemic has greatly increased the magnitude of loneliness among the people suffering from a lack of social connectedness. The COVID-19 pandemic has affected not only social contact due to health safety measures, but also individual, economic, and social resources that help people in becoming socially connected. Specifically, changes in demographic, socioeconomic, psychological, and health-related issues have a diverging effect on people’s loneliness. With previous studies documenting increased loneliness during the pandemic but inconsistent evidence on who became lonely, we conducted this study to reveal detailed evidence of loneliness among men and women of various age groups in Japan.

According to our findings, the majority of respondents were lonely prior to the pandemic, and a significant number became lonely during the pandemic. Our results demonstrate the influence of the pandemic on loneliness which are also consistent with the findings of Khan and Kadoya [2], Weissbourd et al. [11], Ausín et al. [12], and Bu et al. [13]. Importantly, loneliness differs significantly between men and women, as well as between different age groups within the men and women group. Before the pandemic, younger people were lonelier than older people, but during the pandemic, older people became lonelier than younger people. The results are consistent with the findings of Khan and Kadoya [2] and Luchetti et al. [14], but contradict Weissbourd et al. [11], Wickens et al. [17], Bu et al. [13], and Li and Wang [18]. It is noteworthy that elderly men and middle-aged women became the loneliest during the pandemic, despite the fact that loneliness in women was not significantly different between various age groups. Although women became lonelier than men during the pandemic, men of certain age groups were lonelier than women. The results indicate that the pandemic affected people of different ages and genders differently. The difference in loneliness within an age group is sometimes so large that classifying men or women as lonelier as a group appears to be an overgeneralization. Therefore, we attempted to explain the causes of loneliness in men and women of different ages separately.

Since our regression results and previous studies show that women became lonelier during the pandemic [2,13,17,21], we investigated the variables associated with loneliness in men and women of different ages. Our findings reveal a variety of variables associated with loneliness for different age groups, implying that each age group has a unique background for becoming lonely and that combining them together can lead to overgeneralization. For example, having a spouse is positively associated with loneliness in most subsamples of men and women, with the exception of the oldest and youngest women, for whom having a spouse is likely to reduce loneliness. Household assets are negatively associated with loneliness in older women. Financial literacy is positively associated with loneliness in older men and women but not in middle-aged women. Financial literacy is often used as a proxy for rational decision making as well as a tool for saving and making profitable investments [42,43,44,45]. Depression and subjective health status, which have been found to be consistently associated with loneliness in men and women in previous studies [2,49,50,51], have different associations for men and women of different ages. For example, depression has positive association with loneliness in older women but a negative association with loneliness in younger women. Depression, as a mood disorder that eventually reduces social contact, has resulted in loneliness among the elderly women. However, depression did not result in the loneliness of younger women who may have overcome the sense of social exclusion through other means. Subjective health status has a positive association with loneliness in the youngest male group but a negative association with loneliness in middle-aged women. While previous research has shown that poor health status impairs social networking ability and makes people lonely [49,50], the pandemic seems to make younger men lonely despite their good subjective health status. However, age, having children, living alone, education, employment status, household income, and myopic view of the future have a minimal and inconsistent association on specific subsamples, while living in rural areas, future anxiety, and financial satisfaction are not significantly associated with loneliness.

We also examined how changes in the demographic and socioeconomic conditions during the pandemic affected the loneliness of men and women of various ages. Our results show that leaving a job during the pandemic is positively associated with loneliness in middle-aged women. It is understandable that leaving a job increases financial concerns during the pandemic and contributes to loneliness among middle-aged women who are responsible for family maintenance. Changes in subjective health status during the pandemic are negatively associated with loneliness in the middle-aged men and women subsamples, implying that their poor subjective health status during the pandemic contributed to their loneliness. This finding is consistent with that of previous studies [49,50]. Finally, changes in depression are positively associated with loneliness in older men and the youngest women. This result, consistent with that of previous studies [2,51], implies that depression contributes to loneliness.

Overall, our findings show that demographic and socioeconomic factors are not similarly related to loneliness in men and women of different ages. The pandemic appears to have influenced people of different genders and ages differently through various economic, psychological, and social channels causing people to feel lonely in different ways. Given the findings that elderly people became lonelier than others, and that not having a spouse, a lack of household assets, and depression were likely to exacerbate their loneliness, this special group should be supervised by the government so that they do not develop additional physical and mental conditions as a result of loneliness. Given Japan’s ageing society, this is a critical task for the government.

Our study has certain limitations that should be considered when interpreting the results. First, the number of observations in a few subsamples were low, which might have affected the regression results. Although we have used weighted regression to reduce the effect, we cannot rule out the possibility of bias. Second, we had to exclude several observations due to missing values on important socioeconomic variables, which forced us to compromise with the results. Nevertheless, this study provides detailed evidence on the loneliness experienced men and women of different ages during the pandemic. Future studies should be conducted with sufficient and representative samples to investigate the impact of the pandemic on the loneliness of people from various socioeconomic backgrounds in greater depth. Moreover, a longitudinal study on the loneliness among diverse socioeconomic groups should be conducted to understand the direction of change in loneliness and associated factors.

## 5. Conclusions

This study sought to investigate who has become lonely during the pandemic by dividing the population into subsamples based on gender and age, as well as to explain the phenomenon in terms of demographic, socio-economic, and behavioral factors. We demonstrate that loneliness was common among respondents before the pandemic and that a significant number of people became lonely for the first time during the pandemic. Loneliness among younger respondents was generally greater prior to the pandemic, but older people became lonelier during the pandemic. However, loneliness varies significantly across age and gender subsamples. Although depression and subjective health status contributed to loneliness, we found no single explanation for the loneliness of a diverse group of people who became lonely during the pandemic. Subsample analysis reveals that the causes of loneliness differ for each group. Nevertheless, we discovered that older people are at a higher risk of developing loneliness during the pandemic due to a variety of socioeconomic and behavioral issues. Given the prevalence of loneliness among people and the tendency for the number to increase during the pandemic, our study suggests that government and health officials should develop effective remedial measures for specific groups of people rather than a one-size-fits-all policy measure. Given their increased vulnerability to developing further complications, older people should be given special consideration in this regard.

## Figures and Tables

**Table 1:** Variable definitions.

Variables	Definition
Loneliness	Dependent variables The extent to which respondents feel loneliness according to the UCLA methodology. The questions asked to measure respondents’ loneliness were “How often do you feel a lack of companionship”, “How often do you feel left out”, and “How often do you feel isolated from others”. The options to respond to these questions were “Hardly ever or never”, “Some of the time”, and “Often”. Loneliness is a binary variable from the 2021 dataset, where 1 indicates having feelings of loneliness some of the time or often, and 0 = otherwise.
Became lonely	Binary variable: 1 = If a person was having feelings of loneliness some of the time or often in 2021 but not in 2020, and 0 = otherwise
Male *	Explanatory variables Binary variable: 1 = Male and 0 = Female
Age *	Continuous variable: Respondent’s age in 2021
Spouse	Binary variable: 1 = Currently have a spouse or partner and 0 = otherwise
Recently Divorced	Binary variable: 1 = If a person recently got divorced in 2021, and 0 = otherwise
Children	Binary variable: 1 = Having a child/children and 0 = otherwise
Living alone	Binary variable: 1 = Living alone and 0 = Otherwise
Became alone	Binary variable: 1 = If a person recently started living alone in 2021, and 0 = otherwise
Living in rural	Binary variable: 1 = Living in rural areas (not Tokyo special wards or government-designated city areas) and 0 = Otherwise
Education	Discrete variable: Years of education
Employed	Binary variable: 1 = Respondent is employed and 0 = otherwise
Left employment	Binary variable: 1 = If a person recently left an employment in 2021, and 0 = otherwise
Household income	Continuous variable: Annual earned income before taxes and with bonuses of the entire household in 2020 (unit: JPY)
Household assets	Continuous variable: Balance of financial assets (savings, stocks, bonds, insurance, etc.) of entire household (unit: JPY)
Financial literacy *	Continuous variable: Average correct answers to three financial literacy questions
Subjective health status	Ordinal variable: 1 = It does not hold true at all for you; 2 = It is not so true for you; 3 = Neither true nor not true; 4 = It is rather true for you; 5 = It is particularly true for you for the statement “I am now healthy and was generally healthy in the last one year”.
Future anxiety	Ordinal variable: 1 = It does not hold true at all for you; 2 = It is not so true for you; 3 = Neither true nor not true; 4 = It is rather true for you; 5 = It is particularly true for you for the statement “I have anxieties about ‘life after 65 years of age’ (For those who were already aged 65 years or above, ‘life in the future’)”.
Financial satisfaction	Ordinal variable: 1 = Completely disagree; 2 = Disagree; 3 = Neither agree nor disagree; 4 = Agree; 5 = Completely agree, for the statement, “Since the future is uncertain, it is a waste to think about it”. I am happy with my financial status”.
Depression	Ordinal variable: 1 = It does not hold true at all for you; 2 = It is not so true for you; 3 = Neither true nor not true; 4 = It is rather true for you; 5 = It is particularly true for you, for the statement, “I often feel depressed or felt depressed in the last one year”.
Myopic view of the future	Ordinal variable: 1 = Completely disagree; 2 = Disagree; 3 = Neither agree nor disagree; 4 = Agree; 5 = Completely agree with the statement “As the future is uncertain, it is a waste to think about it”.

Note: * indicates data from the 2020 wave.

**Table 2:** Descriptive statistics.

Variable	Mean	Std. Dev.	Min	Max
Dependent variables Loneliness	0.7356	0.4411	0	1
Became lonely	0.1172	0.3217	0	1
Explanatory variables				
Male	0.6692	0.4705	0	1
Age	50.9899	13.6407	21	86
Spouse	0.6703	0.4702	0	1
Children	0.5838	0.4930	0	1
Living alone	0.2011	0.4009	0	1
Living in rural	0.5787	0.4938	0	1
Education	15.0140	2.0943	9	21
Employed	0.6402	0.4800	0	1
Household income *	6.4605	4.1333	0.50	21
Household assets *	21.0000	29.9000	1.25	125
Financial literacy	0.6804	0.3426	0	1
Subjective health status	3.2557	1.0829	1	5
Future anxiety	3.6924	1.1427	1	5
Financial satisfaction	2.7664	1.1144	1	5
Depression	2.9491	1.2167	1	5
Myopic view of the future	2.6740	1.0223	1	5
Observations	3755			

* Unit in million yen.

**Table 3:** Loneliness, stratified by gender and age.

Loneliness	Male				Females				Total
	Age < 36	Age 36–50	Age 51–65	Age > 65	Age < 36	Age 36–50	Age 51–65	Age > 65	
0	64	161	266	207	57	96	87	55	993
	28.07%	20.15%	27.71%	39.35%	15.88%	20.00%	29.19%	52.38%	26.44%
1	164	638	694	319	302	384	211	50	2762
	71.93%	79.85%	72.29%	60.65%	84.12%	80.00%	70.81%	47.62%	73.56%
Total	228	799	960	526	359	480	298	105	3755
	100%	100%	100%	100%	100%	100%	100%	100%	100%
Mean difference	F = 19.87 ***				F = 24.06 ***				
	F = 19.31 ***								

Note: *** *p* < 0.01.

**Table 4:** Became lonely, stratified by gender and age.

Became Lonely	Male				Females				Total
	Age < 36	Age 36–50	Age 51–65	Age > 65	Age < 36	Age 36–50	Age 51–65	Age > 65	
0	199	717	876	455	307	422	247	92	3315
	87.28%	89.74%	91.25%	86.50%	85.52%	87.92%	82.89%	87.62%	88.28%
1	29	82	84	71	52	58	51	13	440
	12.72%	10.26%	8.75%	13.50%	14.48%	12.08%	17.11%	12.38%	11.72%
Total	228	799	960	526	359	480	298	105	3755
	100%	100%	100%	100%	100%	100%	100%	100%	100%
Mean difference	F = 3.11 **				F = 1.39				
	F = 3.27 ***								

Note: *** *p* < 0.01, ** *p* < 0.05.

**Table 5:** Logit regression results of loneliness in 2021 (full sample analysis).

Variables	Dependent Variable: Loneliness			
	Model 1	Model 2	Model 3	Model 4
Male	0.305 *	0.193	0.221	0.281
	(0.170)	(0.169)	(0.180)	(0.192)
Age	−0.0315 ***	−0.0271 ***	−0.0241 ***	−0.0230 ***
	(0.00734)	(0.00733)	(0.00777)	(0.00816)
Spouse	0.116	0.161	0.121	0.180
	(0.200)	(0.197)	(0.199)	(0.230)
Children	−0.531 ***	−0.514 ***	−0.444 ***	−0.416 ***
	(0.149)	(0.149)	(0.162)	(0.157)
Living alone	0.0458	−0.197	−0.0569	0.00684
	(0.220)	(0.228)	(0.231)	(0.253)
Living in rural	−0.0376	−0.0592	−0.100	−0.0717
	(0.152)	(0.145)	(0.153)	(0.158)
Education	0.0203	0.0621	0.0734	0.0735
	(0.0385)	(0.0425)	(0.0451)	(0.0468)
Employed		0.0681	0.0178	0.0243
		(0.142)	(0.144)	(0.150)
Log of HH income		−0.316 ***	−0.261 **	−0.251 **
		(0.101)	(0.109)	(0.112)
Log of HH assets		−0.162 ***	−0.104	−0.133 *
		(0.0575)	(0.0699)	(0.0735)
Financial literacy		0.184	0.121	0.236
		(0.184)	(0.193)	(0.198)
Subjective health			−0.384 ***	−0.283 ***
			(0.0538)	(0.0546)
Future Anxiety			0.333 ***	0.209 ***
			(0.0561)	(0.0624)
Financial satisfaction			0.0381	0.0789
			(0.0774)	(0.0819)
Depression				0.396 ***
				(0.0563)
Myopic view of the future				0.0435
				(0.0576)
Constant	2.269 ***	8.826 ***	6.779 ***	5.566 ***
	(0.574)	(1.625)	(1.775)	(1.900)
Observations	3755	3755	3755	3755
Log likelihood	0.000	0.000	0.000	0.000
Chi^2^ statistics	63.62	78.50	197.8	235.7
*p*-value	0.000	0.000	0.000	0.000

Robust standard errors in parentheses. *** *p* < 0.01, ** *p* < 0.05, * *p* < 0.1.

**Table 6:** Logit regression results of loneliness in 2021 (subsample analysis: gender and age group).

Variables	Dependent Variable: Loneliness							
	Male				Females			
	Age < 36	Age 36–50	Age 51–65	Age > 65	Age < 36	Age 36–50	Age 51–65	Age > 65
Age	0.0595	0.0296	−0.0409 **	0.00280	−0.0237	0.0121	−0.0440	−0.197 **
	(0.0625)	(0.0225)	(0.0200)	(0.0310)	(0.0764)	(0.0307)	(0.0372)	(0.0836)
Spouse	−0.0881	−0.405	−0.110	1.771 **	−0.894	0.215	−0.0630	−0.233
	(0.705)	(0.322)	(0.289)	(0.709)	(0.576)	(0.386)	(0.562)	(1.024)
Children	−1.155 **	0.363	−0.154	−0.670	−0.145	−0.141	−0.645 *	−0.429
	(0.572)	(0.261)	(0.220)	(0.450)	(0.459)	(0.311)	(0.371)	(0.941)
Living alone	−1.417 **	0.562 *	0.0559	1.590 *	−0.257	0.226	−0.0335	1.586
	(0.682)	(0.316)	(0.336)	(0.843)	(0.569)	(0.454)	(0.674)	(1.260)
Living in rural	1.129 ***	0.00320	−0.0935	0.0785	0.232	−0.543 **	−0.151	−0.0943
	(0.420)	(0.191)	(0.163)	(0.245)	(0.299)	(0.248)	(0.307)	(0.531)
Education	0.120	0.0273	−0.0345	−0.0329	0.150	0.0157	−0.0508	0.127
	(0.122)	(0.0483)	(0.0430)	(0.0701)	(0.105)	(0.0721)	(0.0980)	(0.160)
Employed	−0.271	−0.0622	−0.376	−0.231	−0.366	0.0853	0.154	0.0854
	(0.863)	(0.430)	(0.266)	(0.264)	(0.370)	(0.293)	(0.369)	(0.847)
Log of HH income	−0.815 *	−0.109	−0.160	−0.480*	−0.324	−0.227	−0.168	0.113
	(0.437)	(0.203)	(0.157)	(0.268)	(0.410)	(0.236)	(0.254)	(0.591)
Log of HH assets	0.483 **	0.100	0.0770	−0.0875	−0.0666	−0.0932	−0.0563	−0.396
	(0.211)	(0.0872)	(0.0684)	(0.121)	(0.176)	(0.112)	(0.140)	(0.359)
Financial literacy	0.484	−0.0495	−0.218	−0.558	−0.303	0.0397	0.602	1.921 *
	(0.595)	(0.306)	(0.287)	(0.548)	(0.491)	(0.347)	(0.448)	(1.012)
Subjective health	−0.135	−0.282 ***	−0.220 ***	−0.212 *	−0.155	−0.435 ***	−0.238	−0.557 **
	(0.217)	(0.0984)	(0.0780)	(0.121)	(0.164)	(0.128)	(0.163)	(0.278)
Future Anxiety	−0.0679	0.129	0.0733	0.214	−0.0956	0.110	0.130	0.520
	(0.205)	(0.0970)	(0.0798)	(0.150)	(0.185)	(0.120)	(0.156)	(0.356)
Financial satisfaction	−0.119	−0.213 **	−0.0563	−0.00437	0.00110	−0.00405	0.0248	0.961 ***
	(0.205)	(0.105)	(0.0892)	(0.162)	(0.212)	(0.144)	(0.161)	(0.357)
Depression	0.593 ***	0.332 ***	0.617 ***	0.242 *	0.520 ***	0.428 ***	0.574 ***	1.189 ***
	(0.216)	(0.0934)	(0.0816)	(0.135)	(0.160)	(0.118)	(0.151)	(0.414)
Myopic view of the future	0.0446	0.154*	0.0950	0.0557	−0.0164	−0.0501	−0.171	0.620*
	(0.204)	(0.0931)	(0.0817)	(0.129)	(0.177)	(0.134)	(0.141)	(0.362)
Constant	1.794	−0.493	4.583 *	8.137 *	6.501	5.718	6.832 *	7.883
	(6.014)	(2.950)	(2.741)	(4.373)	(5.217)	(3.607)	(4.104)	(9.612)
Observations	228	799	960	526	359	480	298	105
Log likelihood	0.000	0.000	0.000	0.000	0.000	0.000	0.000	0.000
Chi^2^ statistics	41.04	57.97	126.5	43.42	42.19	51.18	49.08	35.10
*p*-value	0.000	0.000	0.000	0.000	0.000	0.000	0.000	0.000

Robust standard errors in parentheses. *** *p* < 0.01, ** *p* < 0.05, * *p* < 0.1.

**Table 7:** Logit regression results of becoming lonely in 2021 (full sample analysis).

Variables	Dependent Variable: Became Lonely			
	Model 5	Model 6	Model 7	Model 8
Male	−0.276 *	−0.368 **	−0.354 **	−0.359 **
	(0.159)	(0.177)	(0.176)	(0.174)
Age	−0.00413	5.73 × 10^−5^	−0.000689	−0.000423
	(0.00732)	(0.00810)	(0.00789)	(0.00790)
Spouse	0.403	0.346	0.340	0.337
	(0.249)	(0.252)	(0.254)	(0.254)
Children	0.0176	0.000215	−0.00159	−0.00380
	(0.167)	(0.170)	(0.171)	(0.171)
Living alone	0.0112	0.0188	−0.000270	0.00210
	(0.252)	(0.263)	(0.264)	(0.263)
Living in rural	−0.174	−0.165	−0.160	−0.163
	(0.165)	(0.164)	(0.161)	(0.161)
Education	0.0269	0.0170	0.0150	0.0126
	(0.0397)	(0.0430)	(0.0420)	(0.0409)
Employed		0.0888	0.101	0.103
		(0.161)	(0.160)	(0.159)
Log of HH income		0.152	0.121	0.120
		(0.127)	(0.125)	(0.124)
Log of HH assets		−0.103	−0.123	−0.127
		(0.0768)	(0.0835)	(0.0823)
Financial literacy		0.297	0.307	0.291
		(0.232)	(0.234)	(0.240)
Subjective health			0.0531	0.0521
			(0.0726)	(0.0744)
Future Anxiety			0.0400	0.0391
			(0.0624)	(0.0655)
Financial satisfaction			0.120	0.123
			(0.0837)	(0.0831)
Depression				−0.00721
				(0.0630)
Myopic view of the future				−0.0507
				(0.0822)
Constant	−2.219 ***	−3.137	−2.968	−2.683
	(0.748)	(1.993)	(1.948)	(1.932)
Observations	3755	3755	3755	3755
Log likelihood	0.000	0.000	0.000	0.000
Chi^2^ statistics	15.16	20.21	22.39	26.74
*p*-value	0.034	0.043	0.071	0.045

Robust standard errors in parentheses. *** *p* < 0.01, ** *p* < 0.05, * *p* < 0.1.

**Table 8:** Logit regression results of becoming lonely in 2021 (subsample analysis: gender and age group).

Variables	Dependent Variable: Became Lonely							
	Male				Females			
	Age < 36	Age 36–50	Age 51–65	Age > 65	Age < 36	Age 36–50	Age 51–65	Age > 65
Age	0.193 ***	−0.00584	0.0289	0.0339	−0.0746	−0.0562	0.0857 *	0.00362
	(0.0571)	(0.0257)	(0.0284)	(0.0606)	(0.0588)	(0.0350)	(0.0467)	(0.107)
Spouse	2.047 **	1.008 **	−0.0895	1.605 **	−1.019 *	0.0885	1.107 *	−2.172 **
	(0.888)	(0.476)	(0.490)	(0.656)	(0.545)	(0.509)	(0.567)	(1.091)
Children	−0.602	−0.299	0.147	0.0189	0.961 **	0.394	−0.352	0.788
	(0.627)	(0.346)	(0.374)	(0.569)	(0.447)	(0.380)	(0.436)	(1.448)
Living alone	1.555	0.423	−0.622	1.010	−0.277	0.916 *	0.215	−0.832
	(0.996)	(0.461)	(0.523)	(0.674)	(0.604)	(0.531)	(0.704)	(1.188)
Living in rural	0.626	−0.138	0.200	−0.361	0.508	−0.257	−0.392	−0.832
	(0.471)	(0.248)	(0.238)	(0.371)	(0.399)	(0.297)	(0.358)	(0.655)
Education	−0.206	−0.0555	−0.0513	−0.0226	−0.117	0.0818	−0.166 *	0.359 *
	(0.134)	(0.0545)	(0.0544)	(0.0993)	(0.113)	(0.0814)	(0.0970)	(0.214)
Employed	0.176	0.585	0.0580	0.108	0.877 **	0.0186	−0.00922	−0.592
	(0.929)	(0.640)	(0.388)	(0.389)	(0.412)	(0.354)	(0.416)	(1.110)
Log of HH income	0.0417	0.0812	−0.0751	0.300	0.398	0.308	0.0452	1.779 **
	(0.532)	(0.304)	(0.207)	(0.303)	(0.347)	(0.281)	(0.234)	(0.864)
Log of HH assets	0.257	0.113	0.0799	−0.143	−0.1000	−0.0385	−0.269 *	−1.103 **
	(0.293)	(0.105)	(0.107)	(0.171)	(0.207)	(0.121)	(0.152)	(0.498)
Financial literacy	−0.262	−0.0426	0.175	1.362 *	−0.216	−0.785 *	0.906 *	2.080
	(0.540)	(0.387)	(0.417)	(0.763)	(0.635)	(0.449)	(0.547)	(1.513)
Subjective health	0.439 *	0.109	0.160	−0.108	0.0980	−0.244 *	0.0652	−0.101
	(0.241)	(0.120)	(0.122)	(0.156)	(0.208)	(0.139)	(0.178)	(0.402)
Future Anxiety	−0.0565	−0.0394	0.0350	0.192	0.280	−0.241	0.0597	−0.336
	(0.234)	(0.120)	(0.146)	(0.200)	(0.176)	(0.150)	(0.171)	(0.421)
Financial satisfaction	0.376	−0.0543	−0.0697	0.399	0.0768	0.0402	0.270	0.153
	(0.289)	(0.131)	(0.156)	(0.244)	(0.172)	(0.149)	(0.185)	(0.492)
Depression	0.0277	−0.185	0.0934	0.121	−0.313 **	−0.216 *	−0.0939	0.945 **
	(0.199)	(0.120)	(0.108)	(0.187)	(0.158)	(0.129)	(0.146)	(0.421)
Myopic view of the future	0.0679	−0.119	−0.0278	−0.300	−0.184	0.179	0.143	0.673 *
	(0.221)	(0.103)	(0.132)	(0.191)	(0.177)	(0.166)	(0.157)	(0.404)
Constant	−13.93 *	−4.379	−4.255	−9.829	−3.070	−3.115	−2.865	−20.08
	(7.681)	(4.189)	(4.038)	(6.247)	(5.868)	(4.067)	(4.100)	(14.42)
Observations	228	799	960	526	359	480	298	105
Log likelihood	0.000	0.000	0.000	0.000	0.000	0.000	0.000	0.000
Chi^2^ statistics	56.32	19.69	11.25	23.22	22.41	20.01	15.95	18.85
*p*-value	0.000	0.184	0.735	0.080	0.097	0.172	0.385	0.221

Robust standard errors in parentheses. *** *p* < 0.01, ** *p* < 0.05, * *p* < 0.1.

**Table 9:** Logit regression results of becoming lonely in 2021 (full sample analysis).

Variables	Dependent Variable: Became Lonely			
	Model 9	Model 10	Model 11	Model 12
Male	−0.279 *	−0.300 *	−0.306 *	−0.265
	(0.158)	(0.161)	(0.161)	(0.163)
Age	−0.00360	−0.00491	−0.00529	−0.00677
	(0.00728)	(0.00728)	(0.00737)	(0.00772)
Recently divorced	−0.849	−0.823	−0.858	−0.818
	(0.893)	(0.895)	(0.877)	(0.815)
Children	0.221	0.226	0.233	0.221
	(0.145)	(0.144)	(0.144)	(0.146)
Became alone	0.226	0.246	0.289	0.220
	(0.771)	(0.778)	(0.768)	(0.707)
Living in rural	−0.161	−0.157	−0.166	−0.157
	(0.164)	(0.166)	(0.164)	(0.172)
Education	0.0335	0.0229	0.0236	0.0213
	(0.0386)	(0.0403)	(0.0392)	(0.0411)
Left employment		−0.0478	−0.0282	−0.0138
		(0.315)	(0.317)	(0.317)
Log of change in household income		0.00809	0.0331	0.0341
		(0.191)	(0.190)	(0.196)
Log of change in household assets		−0.00537	−0.00172	0.0166
		(0.110)	(0.109)	(0.113)
Financial literacy		0.260	0.297	0.300
		(0.212)	(0.210)	(0.213)
Change in health status			−0.0257	0.00397
			(0.0730)	(0.0760)
Change in future anxiety			0.182 ***	0.101
			(0.0683)	(0.0709)
Change in financial satisfaction			−0.000188	0.00254
			(0.0731)	(0.0737)
Change in depression				0.232 ***
				(0.0745)
Change in the myopic view				0.0580
				(0.0615)
Constant	−2.197 ***	−2.139 ***	−2.165 ***	−2.136 ***
	(0.730)	(0.738)	(0.738)	(0.758)
Observations	3755	3755	3755	3755
Log likelihood	0.000	0.000	0.000	0.000
Chi^2^ statistics	9.176	11.33	17.86	35.72
*p*-value	0.240	0.416	0.213	0.003

Robust standard errors in parentheses. *** *p* < 0.01, * *p* < 0.1.

**Table 10:** Logit regression results of becoming lonely in 2021 (subsample analysis: gender and age group).

Variables	Dependent Variable: Became Lonely							
	Male				Females			
	Age < 36	Age 36–50	Age 51–65	Age > 65	Age < 36	Age 36–50	Age 51–65	Age > 65
Age	0.203 ***	−0.0228	0.0301	0.0251	−0.0521	−0.0289	0.0678 *	0.203 ***
	(0.0477)	(0.0274)	(0.0282)	(0.0746)	(0.0527)	(0.0338)	(0.0412)	(0.0477)
Recently divorced			2.506 ***					
			(0.793)					
Children	−0.229	0.269	0.247	0.0138	0.506	0.199	0.230	−0.229
	(0.582)	(0.246)	(0.258)	(0.463)	(0.380)	(0.285)	(0.402)	(0.582)
Became alone		1.036			1.874 **	0.803		
		(0.893)			(0.771)	(1.676)		
Living in rural	0.624	−0.267	0.219	−0.0696	0.621	−0.279	−0.392	0.624
	(0.448)	(0.241)	(0.238)	(0.409)	(0.438)	(0.297)	(0.360)	(0.448)
Education	−0.126	−0.00235	−0.0676	0.00694	−0.0901	0.113	−0.180 *	−0.126
	(0.129)	(0.0490)	(0.0545)	(0.105)	(0.122)	(0.0778)	(0.0964)	(0.129)
Left employment	0.799		−1.196	−0.990	−0.561	1.258**	−0.131	0.799
	(1.270)		(1.055)	(0.967)	(0.699)	(0.591)	(0.927)	(1.270)
Log of change in household income	−0.260	−0.0401	0.00453	−0.871	0.352	−0.260	0.0434	−0.260
	(0.630)	(0.284)	(0.396)	(0.571)	(0.433)	(0.324)	(0.329)	(0.630)
Log of change in household assets	0.231	−0.0843	−0.0948	0.00777	−0.198	0.278	0.0724	0.231
	(0.301)	(0.215)	(0.212)	(0.225)	(0.319)	(0.219)	(0.433)	(0.301)
Financial literacy	−0.0807	0.0737	0.256	1.441*	0.256	−0.619	0.739	−0.0807
	(0.509)	(0.373)	(0.408)	(0.810)	(0.675)	(0.433)	(0.488)	(0.509)
Change in health status	−0.139	−0.211 **	0.0142	−0.0300	0.105	−0.295 **	−0.0157	−0.139
	(0.149)	(0.104)	(0.124)	(0.109)	(0.151)	(0.121)	(0.147)	(0.149)
Change in future anxiety	0.0479	0.163	−0.0149	−0.00511	−0.0741	0.00846	0.205	0.0479
	(0.263)	(0.125)	(0.140)	(0.210)	(0.166)	(0.139)	(0.173)	(0.263)
Change in financial satisfaction	0.215	−0.109	−0.0325	0.176	−0.268 *	−0.147	0.285	0.215
	(0.158)	(0.143)	(0.149)	(0.189)	(0.151)	(0.160)	(0.221)	(0.158)
Change in depression	0.158	0.111	0.321 ***	0.464 ***	0.404 ***	0.104	0.122	0.158
	(0.214)	(0.0959)	(0.117)	(0.161)	(0.152)	(0.136)	(0.153)	(0.214)
Change in the myopic view	0.100	0.108	−0.0531	−0.123	−0.0428	0.0791	0.107	0.100
	(0.177)	(0.122)	(0.116)	(0.206)	(0.134)	(0.124)	(0.147)	(0.177)
Constant	−6.796 ***	−1.238	−3.599 **	−5.032	0.260	−2.218	−3.378	−6.796 ***
	(2.102)	(1.516)	(1.729)	(7.009)	(2.308)	(1.945)	(2.763)	(2.102)
Observations	222	784	952	522	355	477	290	222
Log likelihood	0.000	0.000	0.000	0.000	0.000	0.000	0.000	0.000
Chi^2^ statistics	39.82	17.36	27.84	22.08	28.19	19.34	16.23	39.82
*p*-value	0.000	0.183	0.015	0.054	0.013	0.152	0.237	0.000

Robust standard errors in parentheses. *** *p* < 0.01, ** *p* < 0.05, * *p* < 0.1.

## Data Availability

Data are available upon request.
